# Tenecteplase versus alteplase for intravenous thrombolysis of acute ischemic stroke patients with large-vessel occlusion: a systematic review and meta-analysis

**DOI:** 10.3389/fneur.2025.1487711

**Published:** 2025-03-19

**Authors:** Beibei Yao, Xintong Wang, Yao Wu, Qing Zhu, Li Li, Xiaogang Tang, Minghua Wu

**Affiliations:** Department of Neurology, Affiliated Hospital of Nanjing University of Chinese Medicine, Jiangsu Province Hospital of Chinese Medicine, Nanjing, China

**Keywords:** tenecteplase, alteplase, ischemic stroke, large vessel occlusion, meta-analysis

## Abstract

**Background:**

Tenecteplase (TNK) was found non-inferior to alteplase (ALT) for acute ischemic stroke (AIS). We sought to further elucidate the efficacy and safety of intravenous TNK versus ALT for AIS patients with large-vessel occlusion (LVO).

**Methods:**

We systematically searched PubMed, Embase, Web of Science, and https://clinicaltrials.gov/ till 20 January 2024 for randomized controlled clinical trials (RCTs) comparing TNK with ALT in AIS patients with LVO. The quality of the included studies was estimated using the Cochrane Risk of Bias Tool. Pooled analysis and publication bias were conducted using RevMan 5.3 and Stata 15. Risk ratios (RRs) with 95% confidence intervals (95% CIs) were reported for each outcome measure. The primary outcome was excellent neurological recovery, which was defined as a modified Rankin Scale (mRS) score of 0–1 at 90 days, and safety outcomes included any parenchymal hematoma, sympomatic intracerebral hemorrhage, and 3-month death.

**Results:**

Five RCTs enrolling 1,028 patients were included. There were no significant differences in terms of 90-day excellent neurological recovery (RR 1.18; 95% CI 1.00–1.40; *p* = 0.05), good neurological recovery (RR 1.18; 95% CI 0.90–1.54; *p* = 0.23), early neurological improvement (RR 1.00; 95% CI 0.57–1.77; *p* = 1.00), or successful reperfusion (RR 1.15; 95% CI 0.93–1.44; *p* = 0.20). In addition, no significant differences were observed in safety outcomes, including any parenchymal hematoma (RR 1.01; 95% CI 0.70–1.45; *p* = 0.98), symptomatic intracerebral hemorrhage (RR 1.14; 95% CI 0.62–2.10; *p* = 0.68), or 3-month mortality (RR 1.22; 95% CI 0.52–2.84; *p* = 0.65).

**Conclusion:**

TNK is an alternative to ALT for thrombolysis in AIS patients with confirmed LVO, offering lower cost and easier administration without increasing safety concerns.

**Systematic review registration:**

https://www.crd.york.ac.uk/prospero/, identifier CRD42024540215.

## Introduction

Although intravenous thrombolysis with alteplase was the only approved agent for acute ischemic stroke, accumulating evidence indicates that tenecteplase may represent an alternative for its longer half-life, easier administration, and greater resistance to plasminogen activator inhibitors than alteplase (1, 2).

However, studies on the exact efficacy of tenecteplase and alteplase for treating AIS patients with LVO remain controversial. Campbell et al. (3) found that intravenous thrombolysis using TNK before endovascular thrombectomy was associated with 2-fold higher rates of successful reperfusion and better functional outcomes than ALT. Nevertheless, in a recently published RCT (4) comparing the safety and efficacy of TNK with ALT in LVO stroke patients who underwent thrombectomy, no difference was detected in successful reperfusion rates in the first and final angiograms. Therefore, we conducted this meta-analysis to compare the efficacy and safety of TNK versus ALT for treating AIS patients with LVO.

## Methods

This study was performed following the prespecified protocol according to the Preferred Reporting Items for Systematic Reviews and Meta-Analyses (PRISMA) guidelines.

### Data sources and inclusion and exclusion criteria

Two investigators (XW and YW) performed literature searches independently in PubMed, Embase, Web of Science, Cochrane Library, and ClinicalTrials.gov from inception to 20 January 2024. The search strategy included keywords such as “tenecteplase,” “alteplase,” “stroke,” “cerebral infarction,” “brain ischemia,” and “Randomized Controlled Trial.” Only studies published in the English language were searched. All references from the included studies and previous relevant systematic reviews were manually searched as well.

The inclusion criteria were as follows: (1) randomized clinical trial, (2) thrombolysis with TNK versus ALT, and (3) AIS patients with LVO.

The exclusion criteria for this meta-analysis were as follows: (1) a follow-up time of no more than 90 days; (2) incomplete clinical trials; (3) non-English studies; (4) basic experimental research, case reports, conference abstracts, and reviews; and (5) incomplete data and repeated publications.

### Data extraction and outcomes

Data were extracted and documented by XW and YW. QZ and LL verified the extracted data. Details recorded from each study included the name of the study, study period, country, sample size, TNK dose(s), mean age, baseline NIHSS, occluded vessel, endovascular thrombectomy (%), and primary outcome. Any disagreements were resolved by consensus review.

The primary outcome was excellent neurological recovery at 90 days, defined as an mRS score of 0–1. The secondary outcomes were as follows: (1) good neurological recovery at 90 days (defined as an mRS ≤ 2), (2) early neurological improvement (according to the definition used in each study), (3) successful reperfusion (defined as an eTICI score of 2b-3), (4) any parenchymal hematoma (any PH), (5) symptomatic intracranial hemorrhage (sICH, according to the definition used in each study), and (6) 3-month mortality.

### Quality assessment

YW and QZ independently conducted the quality evaluation according to the Cochrane Collaboration tool (5) in seven domains, including random sequence generation, allocation concealment, blinding of participants and personnel, blinding of outcome assessment, selective reporting, incomplete outcome data, and other biases. Arguments were settled by consensus. RevMan 5.3 software was used to visualize the plot of risk bias.

### Statistics

Data synthesis and statistical analysis were conducted using STATA 15 (StataCorp, USA) and Review Manager 5.3 (RevMan; Cochrane Collaboration). For dichotomous variables, risk ratios (RRs) and 95% confidence intervals (CIs) were pooled, and a *p*-value of ≤0.05 was considered statistically significant. The I^2^ value was a quantitative measure to assess the heterogeneity. A fixed effects model was used if I^2^ ≤ 50%, suggesting a small heterogeneity among the included studies (6). Otherwise, a random-effects model was used. Moreover, sensitivity analysis was conducted to assess the quality and consistency of the results. Publication bias of the included RCTs was evaluated using the funnel plot, Egger’s test (7), and Begg’s test (8).

## Results

### Study selection and study characteristics

**Figure 1:**
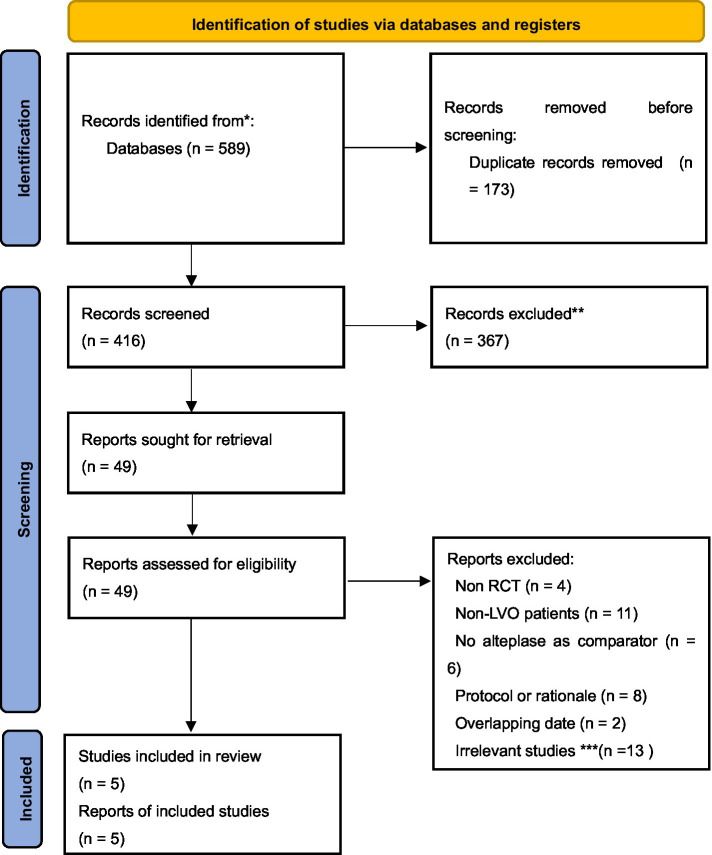
PRISMA flowchart of literature search and study selection.

We identified 589 potentially relevant citations from PubMed, EMBASE, Web of Science, and the Cochrane Library (Figure 1). A total of 173 duplicated articles were excluded from the study. We screened the title and abstract to exclude 367 unrelated articles. Among the remaining 49 studies, 44 were excluded after reading the full article and 5 studies with 1,028 patients met the selection criteria (3, 4, 9–11). However, six of these studies were excluded—three RCTs (12–14) and three pooled analyses (15–17) included no intravenous alteplase treatment control group. In addition, another two articles (18, 19) including overlapping participant data were excluded. The patients were randomly assigned to TNK at doses of 0.1, 0.25, and 0.4 mg/kg and ALT at a standard dose of 0.9 mg/kg. The characteristics of the included studies are presented in Table 1.

**Table 1:** Characteristics of the included studies.

Study	Study period	Country	No. patients (TNK/ALT)	TNK dose(s), mg/kg	Mean age	Baseline NIHSS	Occluded vessel (%)	EVT (%)	Time window
AcT	2019–2022	Canada	263/257	0.25	TNK 74 (65–84) ALT 73 (63–83)	TNK 17 (11–22) ALT 17 (12–22)	ICA 26.0% M1-MCA 45.6% M2-MCA 22.5% BA 6.0%	TNK78.7% ALT 78.2%	4.5 h
ATTEST + Australian TNK $	2008–2013	Australia and Scotland	60/53	0.25	-	-	ICA: 0.9% MCA: 87.1% ACA/PCA: 12.0%	N/A	4.5/6 h
Australian TNK	2008–2011	Australia	50/25	0.1/0.25	TNK(0.1) 72 ± 6.9 TNK(0.25) 68 ± 9.4 ALT 70 ± 8.4	TNK(0.1) 14.5 ± 2.3 TNK(0.25) 14.6 ± 2.3 ALT 14.0 ± 2.3	ACA: 1.3% M1-MCA: 76.0% M2-MCA: 13.3% PCA: 4.0% ACA: 1.3% None: 4.0%	0%	6 h
EXTEND-IA TNK	2015–2017	Australia and New Zealand	101/101	0.25	TNK 70.4 ± 15.1 ALT 71.9 ± 13.7	TNK 17 (12–22) ALT 17 (12–22)	ICA: 23.8% BA: 3.0% M1-MAC: 58.9% M2-MCA: 14.4%	N/A	4.5 h
NOR-TEST*	2012–2016	Norway	52/66	0.4	-	-	ICA: 6.8% M1-MCA: 51.7% M2-MCA: 41.5%	TNK 9.2% ALT 10.3%**	4.5 h

$ Patients with partial or complete vessel occlusion in ATTEST and Australian TNK. *As provided in the Supplement of the relevant publication. ** Of patients with moderate and severe stroke on admission (baseline NIHSS score more than 6).

### Risk of bias and quality assessment

**Figure 2:**
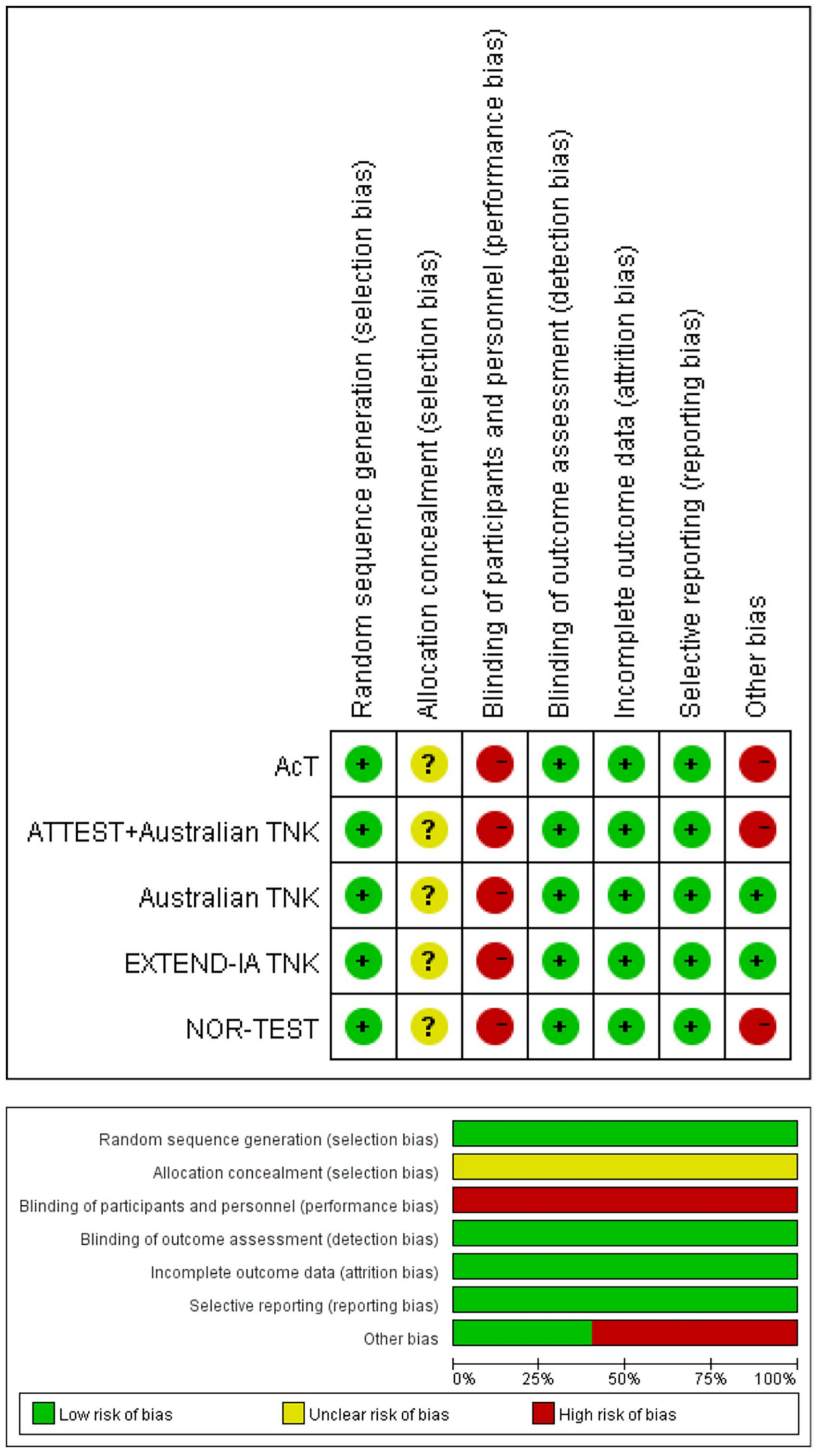
Quality assessment of the risk of bias for each included study.

Assessment for the risk of bias is summarized in Figure 2. All the included trials had a low risk of random sequence generation bias and an unclear risk of allocation concealment. In the domain of the blinding of participants and personnel, all five publications showed a high risk of bias due to different administrations of TNK and ALT. However, although participants were aware of the interventions, the evaluation personnel were not aware. The risk of other bias was marked as high in three studies reporting analyses on subgroups of patients with LVO randomized within the original RCTs.

## Primary outcome

### Excellent neurological recovery (mRS 0–1)

**Figure 3:**
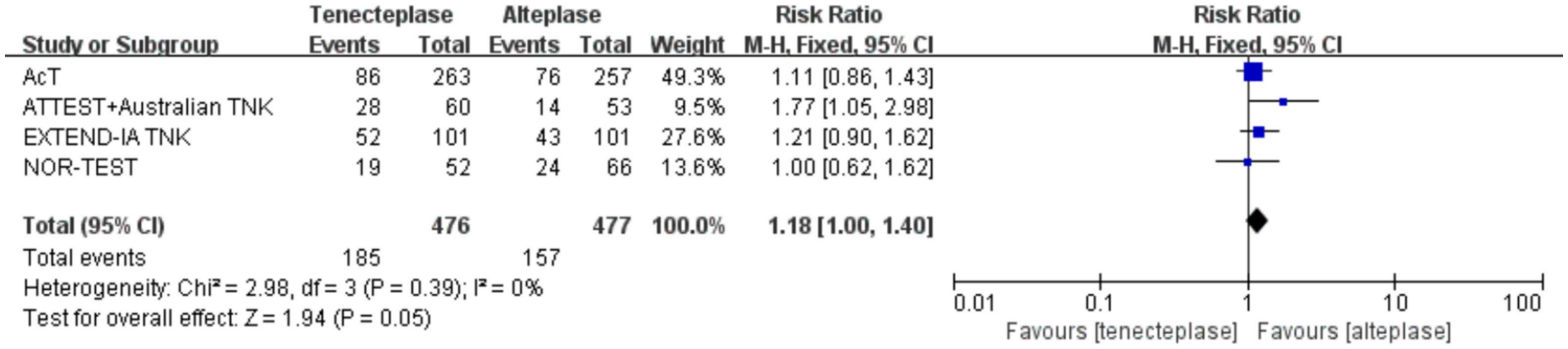
Forest plot of excellent neurological recovery.

For the analysis of mRS score 0–1, 4 studies and 953 patients were included (Figure. 3). Intravenous TNK was superior to ALT (RR 1.18; 95% CI 1.00–1.40; *p* = 0.05) for achieving excellent neurological recovery, although this difference did not reach statistical significance. No significant heterogeneity was observed (*I*^2^ = 0%, *p* = 0.39).

### Secondary outcomes

The pooled results of three studies showed that the rates of 90-day good neurological outcomes were not statistically different between patients receiving TNK and ALT treatments (RR 1.18; 95% CI 0.90–1.54; *p* = 0.23). There was no significant difference in the incidence of early neurological improvement between the intravenous TNK and the ALT treatments (RR 1.00; 95% CI 0.57–1.77; *p* = 1.00). A higher incidence of achieving successful reperfusion was observed in the tenecteplase group than in the alteplase group; however, this difference did not reach statistical significance (RR 1.15; 95% CI 0.93–1.44; *p* = 0.20). In addition, there were no statistical differences between the two groups in the rates of any PH (RR 1.01; 95% CI 0.70–1.45; *p* = 0.98), sICH (RR 1.14; 95% CI 0.62–2.10; *p* = 0.68), or 3-month mortality (RR 1.22; 95% CI 0.52–2.84; *p* = 0.65). We summarized all the pooled results of the primary and secondary outcomes in Table 2.

**Table 2:** Pooled results of primary and secondary outcomes.

	No. of studies	Total no patients	TNK	ALT	Pooled results			Heterogeneity	
					RR	95% CI	*P-*value	I^2^, %	*P*-value
Primary outcome									
Excellent neurological recovery	4	953	476	477	1.18	[1.00–1.40]	**0.05**	0	0.39
Secondary outcomes									
Good neurological recovery	3	797	414	383	1.18	[0.90–1.54]	0.23	67	0.05
Early neurological improvement	3	395	203	192	1.00	[0.57–1.77]	1.00	75	**0.02**
Successful reperfusion	5	1,321	670	651	1.15	[0.93–1.44]	0.20	80	**0.0004**
Any PH	4	903	462	441	1.01	[0.70–1.45]	0.98	24	0.27
Symptomatic ICH	4	903	464	446	1.14	[0.62–2.10]	0.68	0	0.51
3-month mortality	4	903	464	446	1.22	[0.52–2.84]	0.65	79	**0.003**

Bold values are used to highlight that he difference is statistically significant.

### Results of subgroup analysis

**Figure 4:**
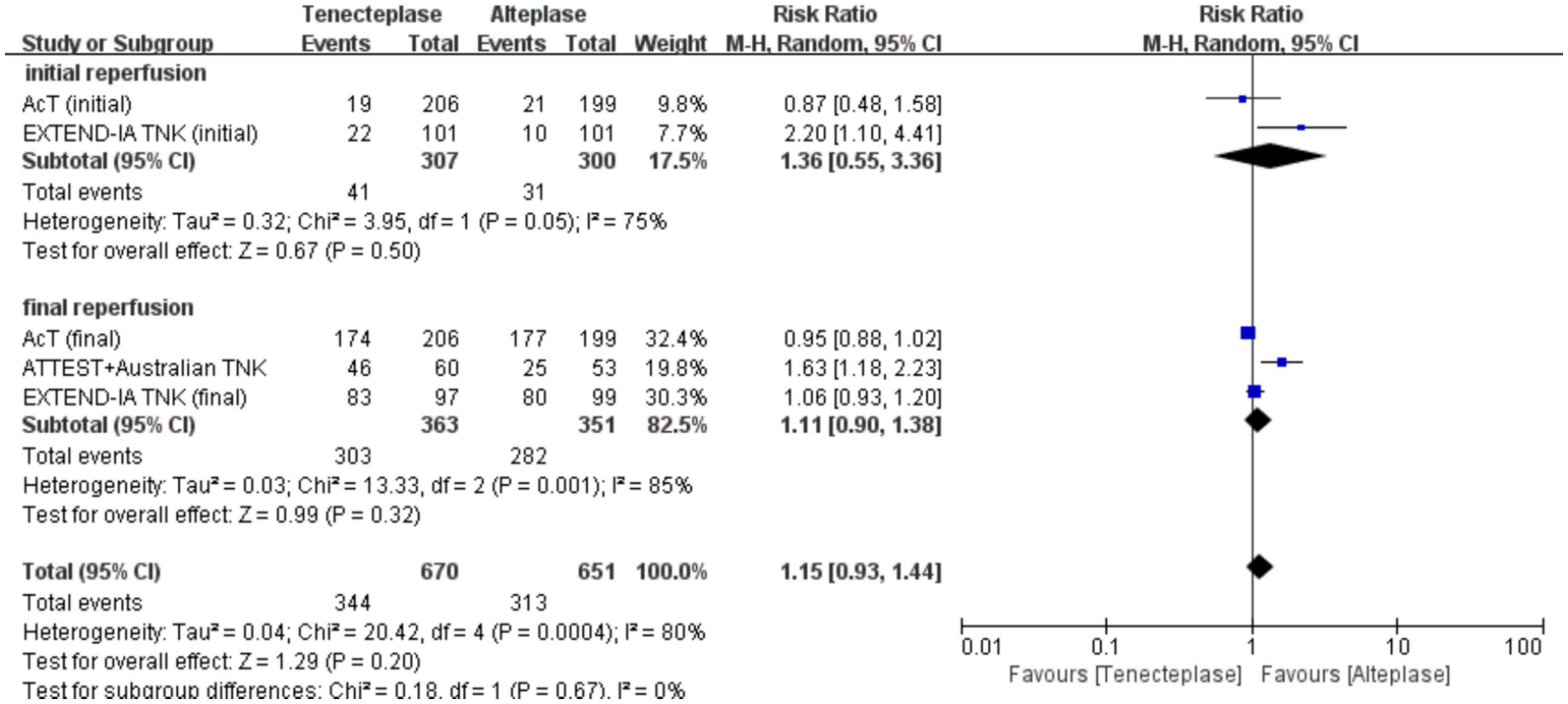
Forest plot of the subgroup analysis of successful reperfusion.

With regard to neuroimaging parameters, no difference was observed in successful reperfusion in the initial (RR 1.36; 95% CI 0.55–3.36; *p* = 0.50) and final (RR 1.11; 95% CI 0.90–1.38; *p* = 0.32) angiographic acquisition. Detailed information regarding the results of the subgroup analysis is provided in Figure 4.

### Sensitivity analysis

Sensitivity analysis was performed using the method of removing item by item to test the stability of the meta-analysis. The findings indicated that the stability of the results had no significant change (Supplementary Figures S1–S7).

### Publication bias

Based on the five included studies, we tested for publication bias in each trial (Supplementary Figures S8–S14). The funnel plot results showed that the left and right sides were symmetrical. Both Begg’s test and Egger’s test results showed symmetry of the results (*p* > 0.05), indicating that there was no publication bias in each study.

## Discussion

Our findings demonstrated that although patients with LVO receiving TNK had higher rates of excellent neurological recovery (RR 1.18; 95% CI 1.00–1.40; *p* = 0.05), the difference was not statistically significant. A numerically higher but statistically non-significant reperfusion was seen on both tenecteplase and alteplase (RR 1.15; 95% CI 0.93–1.44; *p* = 0.20). Further subgroup analysis showed that rates of successful reperfusion at either first or final angiographic acquisition were similar across different groups (RR 1.36; 95% CI 0.55–3.36; *p* = 0.50, RR 1.11; 95% CI 0.90–1.38; *p* = 0.32). Katsanos et al. (20) found that AIS patients with LVO receiving intravenous thrombolysis with TNK have 3-fold higher odds of successful recanalization, which did not corroborate our findings. Previous analysis only included two small sample size trials with a total of 315 patients. However, the latest Intravenous tenecteplase compared with alteplase for acute ischaemic stroke in Canada (AcT) (4) trial including 520 AIS patients with confirmed LVO was added to our study. AcT (4) study found that intravenous tenecteplase conferred similar reperfusion compared to alteplase among patients with LVO. In contrast, the EXTEND-IA TNK (3) trial revealed that intravenously administered tenecteplase was superior in improving early reperfusion among AIS patients before endovascular treatment. We presumed that it could be attributed to the fact that AcT (4) was a pragmatic trial evaluating the safety and efficacy in all AIS patients, while EXTEND-IA TNK (3) only enrolled LVO patients eligible to undergo thrombectomy.

With similar reperfusion, tenecteplase has the advantages of lower price, longer half-time, greater fibrin specificity, and higher resistance to plasminogen activator inhibitors (1, 2). These pharmacological properties allow a single bolus administration, leading to stronger clot dissolution and faster vessel recanalization. The clinical benefit of TNK makes it an alternative drug, especially for AIS patients with LVO, who require rapid transfer for early endovascular thrombectomy in the emergency department or an ambulance. Bivard et al. (21) evaluated the volume of the perfusion lesion in patients receiving thrombolytic treatment in the prehospital mobile stroke units. Compared with alteplase, administration with tenecteplase resulted in a smaller perfusion lesion volume and greater early clinical recovery, providing strong support for the early use of tenecteplase in mobile stroke units. In terms of safety outcomes, there were no significant differences in any PH (RR 1.01; 95% CI 0.70–1.45; *p* = 0.98), sICH (RR 1.14; 95% CI 0.62–2.10; *p* = 0.68), and mortality at 90 days (RR 1.22; 95% CI 0.52–2.84; *p* = 0.65) between TNK and ALT. The more rapidly administered TNK may provide greater practical benefit without increasing the safety concern.

### Limitations

Several limitations should be acknowledged in our meta-analysis. First, subgroups of patients with confirmed LVO from three of the included trials were enrolled. It is well known that subgroup analysis suffers from low power. Second, it is important to acknowledge the apparent overlap in patient enrollment between the ATTEST + Australian TNK and the Australian TNK trials, particularly concerning the 0.25 mg/kg TNK dosage. However, upon further investigation in Table 2, we found that no duplicate data were calculated, thus having little impact on our study outcomes. Third, although the standard dose of alteplase (0.9 mg/kg) was used, the dosage of TNK varied between 0.1, 0.25, and 0.4 mg/kg across the studies. Compared with 0.25 mg/kg, intravenous tenecteplase of 0.4 mg/kg failed to improve cerebral reperfusion before endovascular therapy among patients with large-vessel occlusion ischemic stroke (12). Meanwhile, NOR-TEST 2 part A (22) using a dose of 0.4 mg/kg TNK was prematurely terminated for high intracranial hemorrhage rates. A recently published network meta-analysis (23) including 11 RCTs indicated that tenecteplase 0.25 mg/kg was superior to alteplase 0.9 mg/kg in excellent functional recovery without increased risk of safety outcomes. Fourth, despite included studies evaluating patients eligible for intravenous thrombolysis within 6 h after symptom onset, the findings of this report cannot be extended to patients beyond 6 h or wake-up stroke. A new randomized clinical trial TIMELESS (24) has reported that 0.25 mg/kg tenecteplase could improve recanalization compared with placebo among stroke patients with M1 occlusion between 4.5 and 24 h. In Chinese patients with acute large/medium vessel occlusion or severe stenosis in the anterior circulation and a favorable penumbral profile between 4.5 and 24 h, both tenecteplase 0.25 and 0.32 mg/kg demonstrated promising efficacy and safety (25).

In addition to intravenous thrombolysis, direct thrombectomy or bridging thrombectomy has a significant impact on the prognosis of patients with large-vessel occlusion. Apart from the differences in the time window and tenecteplase dose, no consensus was reached on the definition of outcomes in the included studies (i.e., early neurological improvement and sICH). Otherwise, the analysis is limited to published literature and does not include unpublished studies, ongoing trials, or sub-analyses on LVOs that were not reported separately in publications. Finally, heterogeneity was found across the included outcomes, which limited confidence in our conclusions.

## Conclusion

Our meta-analysis elucidated that TNK may serve as an alternative for thrombolysis without increasing safety concerns. Its quicker administration and lower cost make TNK a viable option for patients with LVO ischemic stroke.

## Data Availability

The original contributions presented in the study are included in the article/Supplementary material, further inquiries can be directed to the corresponding authors.

## References

[1] Tanswell P Modi N Combs D Danays T. Pharmacokinetics and pharmacodynamics of Tenecteplase in fibrinolytic therapy of acute myocardial infarction. Clin Pharmacokinet. (2002) 41:1229–45. doi: 10.2165/00003088-200241150-00001, PMID: 12452736

[2] Warner JJ Harrington RA Sacco RL Elkind MSV. Guidelines for the early Management of Patients with acute ischemic stroke: 2019 update to the 2018 guidelines for the early Management of Acute Ischemic Stroke. Stroke. (2019) 50:3331–2. doi: 10.1161/strokeaha.119.027708 31662117

[3] Campbell BCV Mitchell PJ Churilov L Yassi N Kleinig TJ Dowling RJ . Tenecteplase versus Alteplase before Thrombectomy for ischemic stroke. N Engl J Med. (2018) 378:1573–82. doi: 10.1056/NEJMoa1716405, PMID: 29694815

[4] Bala F Singh N Buck B Ademola A Coutts SB Deschaintre Y . Safety and efficacy of Tenecteplase compared with Alteplase in patients with large vessel occlusion stroke: a Prespecified secondary analysis of the act randomized clinical trial. JAMA Neurol. (2023) 80:824–32. doi: 10.1001/jamaneurol.2023.2094, PMID: 37428494 PMC10334294

[5] Higgins JP Altman DG Gøtzsche PC Jüni P Moher D Oxman AD . The Cochrane Collaboration's tool for assessing risk of Bias in randomised trials. BMJ. (2011) 343:d5928. doi: 10.1136/bmj.d5928, PMID: 22008217 PMC3196245

[6] Higgins JP Thompson SG Deeks JJ Altman DG. Measuring inconsistency in Meta-analyses. BMJ. (2003) 327:557–60. doi: 10.1136/bmj.327.7414.557, PMID: 12958120 PMC192859

[7] Egger M Davey Smith G Schneider M Minder C. Bias in Meta-analysis detected by a simple. Graph Test BMJ. (1997) 315:629–34. doi: 10.1136/bmj.315.7109.629, PMID: 9310563 PMC2127453

[8] Begg CB Mazumdar M. Operating characteristics of a rank correlation test for publication Bias. Biometrics. (1994) 50:1088–101. doi: 10.2307/2533446, PMID: 7786990

[9] Bivard A Huang X Levi CR Spratt N Campbell BCV Cheripelli BK . Tenecteplase in ischemic stroke offers improved recanalization: analysis of 2 trials. Neurology. (2017) 89:62–7. doi: 10.1212/wnl.0000000000004062, PMID: 28576782

[10] Kvistad CE Novotny V Kurz MW Rønning OM Thommessen B Carlsson M . Safety and outcomes of Tenecteplase in moderate and severe ischemic stroke. Stroke. (2019) 50:1279–81. doi: 10.1161/strokeaha.119.025041, PMID: 31009339

[11] Parsons M Spratt N Bivard A Campbell B Chung K Miteff F . A randomized trial of Tenecteplase versus Alteplase for acute ischemic stroke. N Engl J Med. (2012) 366:1099–107. doi: 10.1056/NEJMoa1109842, PMID: 22435369

[12] Campbell BCV Mitchell PJ Churilov L Yassi N Kleinig TJ Dowling RJ . Effect of intravenous Tenecteplase dose on cerebral reperfusion before Thrombectomy in patients with large vessel occlusion ischemic stroke: the Extend-Ia Tnk part 2 randomized clinical trial. JAMA. (2020) 323:1257–65. doi: 10.1001/jama.2020.1511, PMID: 32078683 PMC7139271

[13] Coutts SB Dubuc V Mandzia J Kenney C Demchuk AM Smith EE . Tenecteplase-tissue-type plasminogen activator evaluation for minor ischemic stroke with proven occlusion. Stroke. (2015) 46:769–74. doi: 10.1161/strokeaha.114.008504, PMID: 25677596

[14] Roaldsen MB Eltoft A Wilsgaard T Christensen H Engelter ST Indredavik B . Safety and efficacy of Tenecteplase in patients with wake-up stroke assessed by non-contrast Ct (twist): a multicentre, open-label. Rand Cont Trial Lancet Neurol. (2023) 22:117–26. doi: 10.1016/s1474-4422(22)00484-7, PMID: 36549308

[15] Sarraj A Albers GW Blasco J Arenillas JF Ribo M Hassan AE . Thrombectomy versus medical Management in Mild Strokes due to large vessel occlusion: exploratory analysis from the Extend-Ia trials and a pooled international cohort. Ann Neurol. (2022) 92:364–78. doi: 10.1002/ana.26418, PMID: 35599458

[16] Yogendrakumar V Churilov L Mitchell PJ Kleinig TJ Yassi N Thijs V . Safety and efficacy of Tenecteplase and Alteplase in patients with tandem lesion stroke: a post hoc analysis of the Extend-Ia Tnk trials. Neurology. (2023) 100:e1900–11. doi: 10.1212/wnl.0000000000207138, PMID: 36878701 PMC10159769

[17] Yogendrakumar V Churilov L Mitchell PJ Kleinig TJ Yassi N Thijs V . Safety and efficacy of Tenecteplase in older patients with large vessel occlusion: a pooled analysis of the Extend-Ia Tnk trials. Neurology. (2022) 98:e1292–301. doi: 10.1212/wnl.0000000000013302, PMID: 35017305

[18] Bala F Almekhlafi M Singh N Alhabli I Ademola A Coutts SB . Safety and efficacy of Tenecteplase versus Alteplase in stroke patients with carotid tandem lesions: results from the act trial. Int J Stroke. (2024) 19:322–30. doi: 10.1177/17474930231205208, PMID: 37731173 PMC10903116

[19] Ferguson E Yadav K. Intravenous Tenecteplase compared with Alteplase for acute ischemic stroke in Canada (act): a pragmatic, multicentre, open-label, registry-linked, randomised, controlled. Non Infer Trial Cjem. (2023) 25:121–2. doi: 10.1007/s43678-022-00432-8, PMID: 36577932

[20] Katsanos AH Safouris A Sarraj A Magoufis G Leker RR Khatri P . Intravenous thrombolysis with Tenecteplase in patients with large vessel occlusions: systematic review and Meta-analysis. Stroke. (2021) 52:308–12. doi: 10.1161/strokeaha.120.030220, PMID: 33272127

[21] Bivard A Zhao H Churilov L Campbell BCV Coote S Yassi N . Comparison of Tenecteplase with Alteplase for the early treatment of Ischaemic stroke in the Melbourne Mobile stroke unit (Taste-a): A phase 2, randomised. Open Label Trial Lancet Neurol. (2022) 21:520–7. doi: 10.1016/s1474-4422(22)00171-5, PMID: 35525251

[22] Kvistad CE Næss H Helleberg BH Idicula T Hagberg G Nordby LM . Tenecteplase versus Alteplase for the Management of Acute Ischaemic Stroke in Norway (nor-test 2, part a): a phase 3, randomised, open-label, blinded endpoint. Non Infer Trial Lancet Neurol. (2022) 21:511–9. doi: 10.1016/s1474-4422(22)00124-7, PMID: 35525250

[23] Liang H Wang X Quan X Chen S Qin B Liang S . Different doses of Tenecteplase vs. Alteplase for acute ischemic stroke within 4.5 hours of symptom onset: a network Meta-analysis of randomized controlled trials. Front Neurol. (2023) 14:1176540. doi: 10.3389/fneur.2023.1176540, PMID: 37333014 PMC10274135

[24] Albers GW Jumaa M Purdon B Zaidi SF Streib C Shuaib A . Tenecteplase for stroke at 4.5 to 24 hours with perfusion-imaging selection. N Engl J Med. (2024) 390:701–11. doi: 10.1056/NEJMoa2310392, PMID: 38329148

[25] Cheng X Hong L Churilov L Lin L Ling Y Zhang J . Tenecteplase thrombolysis for stroke up to 24 hours after onset with perfusion imaging selection: the umbrella phase Iia Chablis-T randomised clinical trial. Stroke Vasc Neurol. (2024) 9:551–9. doi: 10.1136/svn-2023-002820, PMID: 38286484 PMC11732838

