# Supplementary material for: Tenecteplase versus alteplase for intravenous thrombolysis of acute ischemic stroke patients with large-vessel occlusion: a systematic review and meta-analysis
Source: Front Neurol. 2025 Mar 19;16:1487711. doi: 10.3389/fneur.2025.1487711 (PMC11963696; doi:10.3389/fneur.2025.1487711)
Supplement: Supplementary file 1 [file Table_1.DOCX]

**Supplemental figure to tenecteplase versus alteplase for intravenous thrombolysis of acute ischemic stroke patients with large vessel occlusion: a systematic review and meta-analysis**


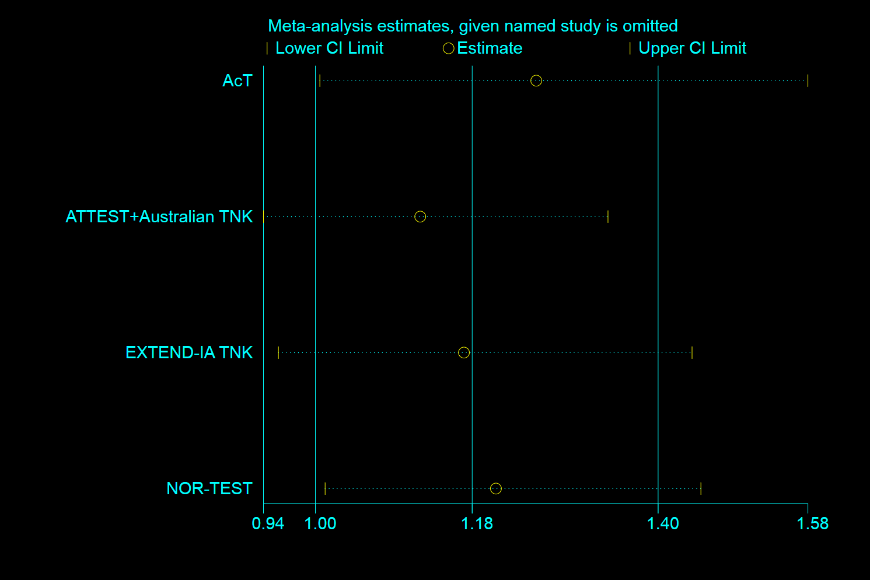


**FIGURE S1** Sensitivity analysis for the comparison of TNK versus ALT for excellent neurological recovery


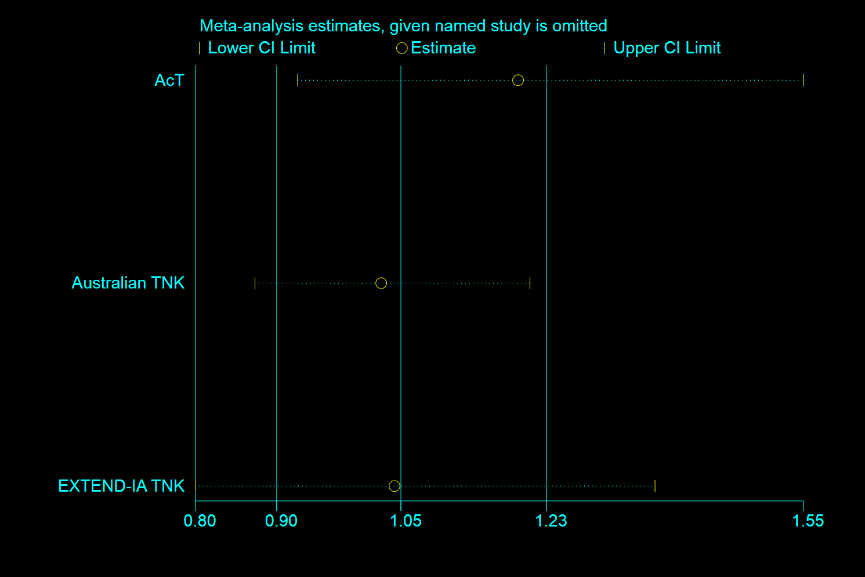


**FIGURE S2** Sensitivity analysis for the comparison of TNK versus ALT for good neurological recovery


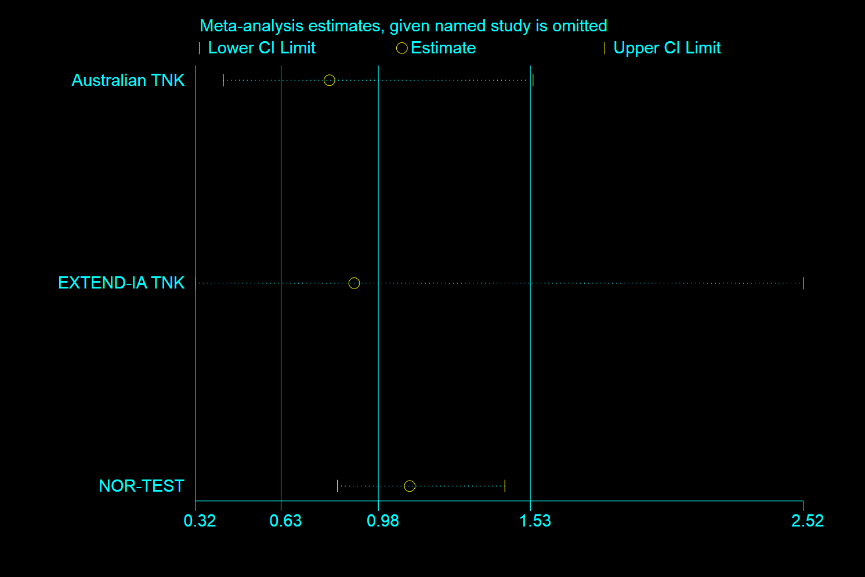


**FIGURE S3** Sensitivity analysis for the comparison of TNK versus ALT for early neurological improvement


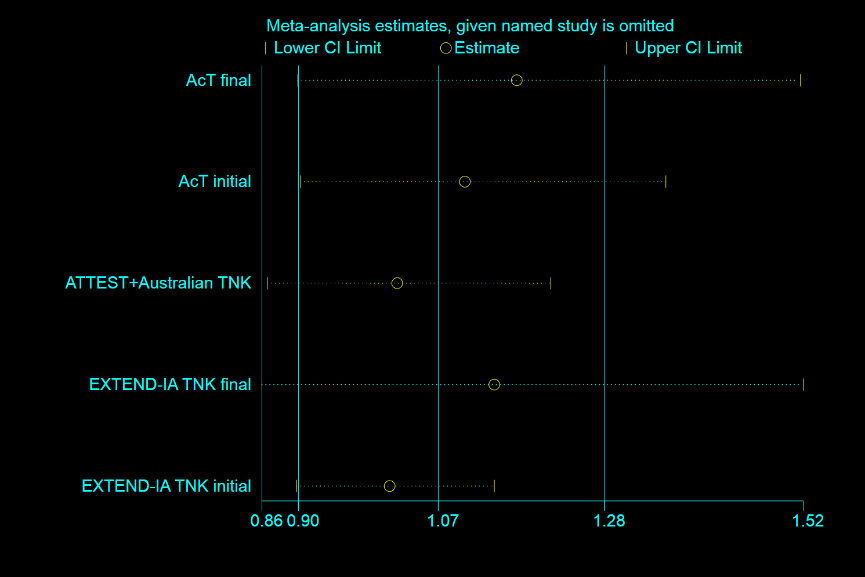


**FIGURE S4** Sensitivity analysis for the comparison of TNK versus ALT for successful reperfusion


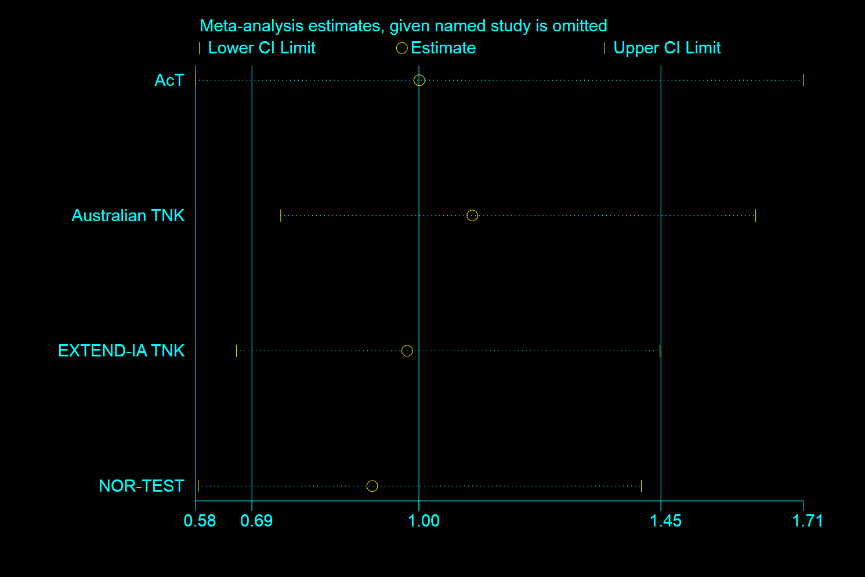


**FIGURE S5** Sensitivity analysis for the comparison of TNK versus ALT for any parenchymal hematoma


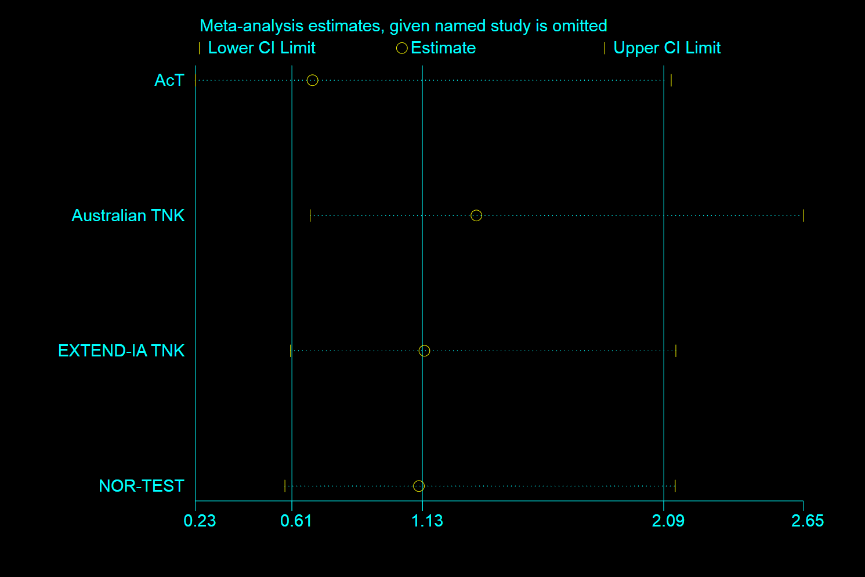


**FIGURE S6** Sensitivity analysis for the comparison of TNK versus ALT for symptomatic intracranial hemorrhage


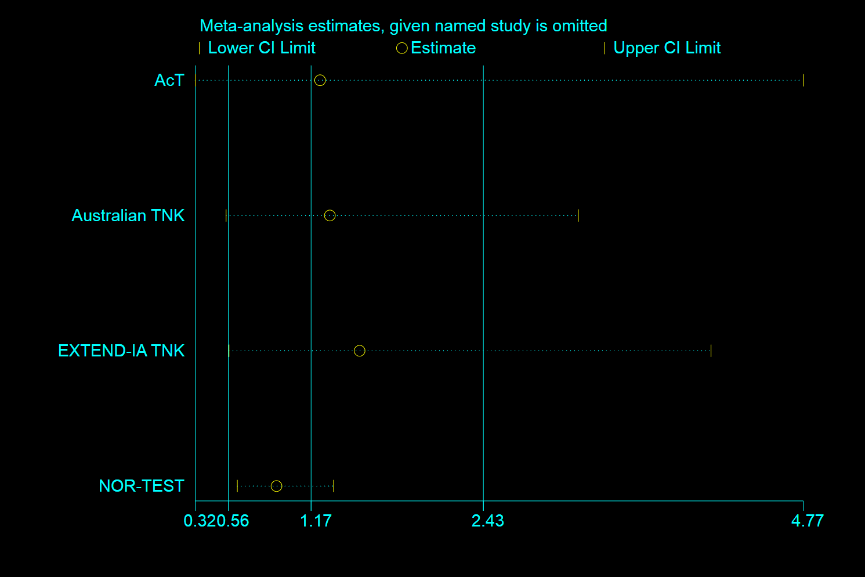


**FIGURE S7** Sensitivity analysis for the comparison of TNK versus ALT for 3-month mortality
