# Supplementary material for: Tenecteplase versus alteplase for intravenous thrombolysis of acute ischemic stroke patients with large-vessel occlusion: a systematic review and meta-analysis
Source: Front Neurol. 2025 Mar 19;16:1487711. doi: 10.3389/fneur.2025.1487711 (PMC11963696; doi:10.3389/fneur.2025.1487711)

**Supplemental figure to tenecteplase versus alteplase for intravenous thrombolysis of acute ischemic stroke patients with large vessel occlusion: a systematic review and meta-analysis**

**FIGURE S8** Funnel plot and Egger’s and Begg’s tests for excellent neurological recovery

**
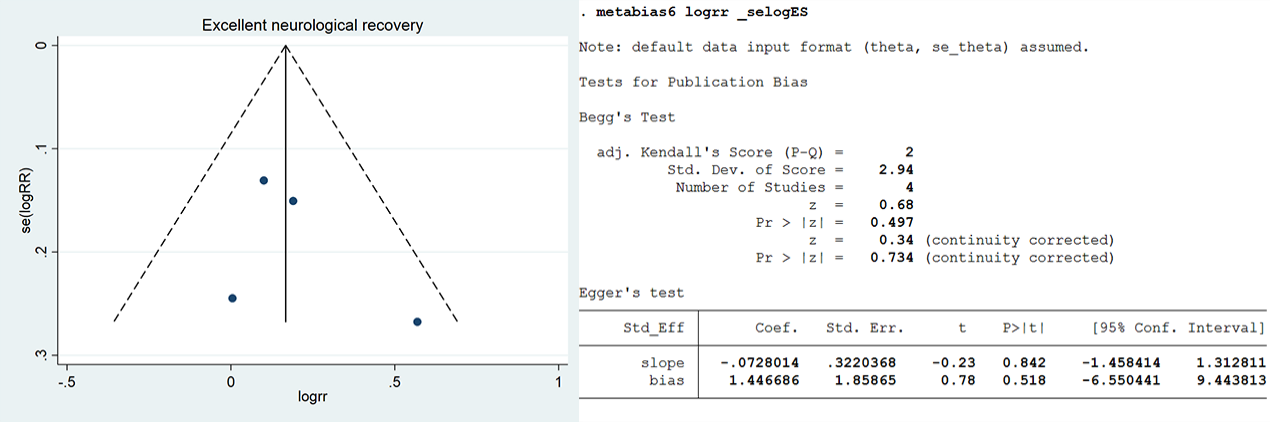
**

**FIGURE S9** Funnel plot and Egger’s and Begg’s tests for good neurological recovery


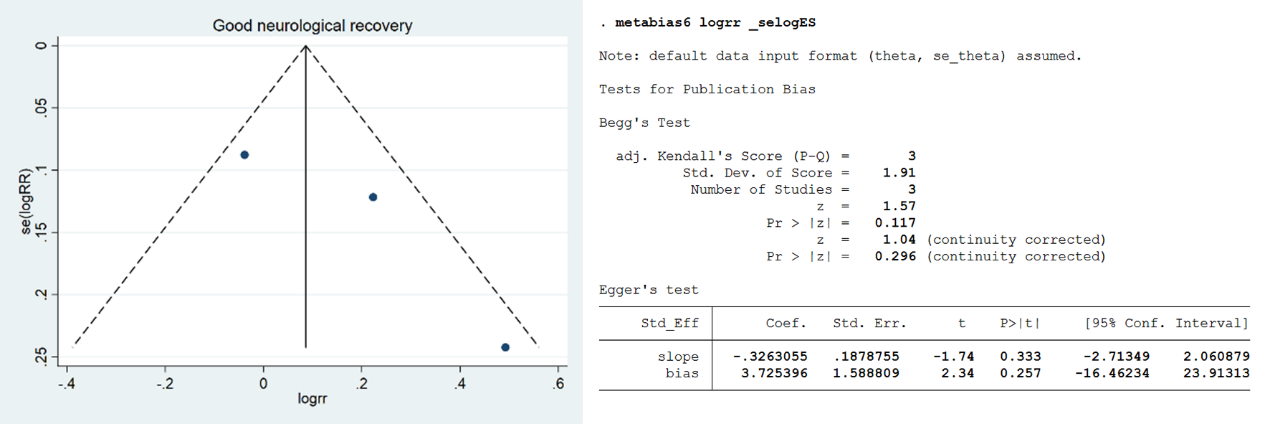


**FIGURE S10** Funnel plot and Egger’s and Begg’s tests for early neurological improvement

**
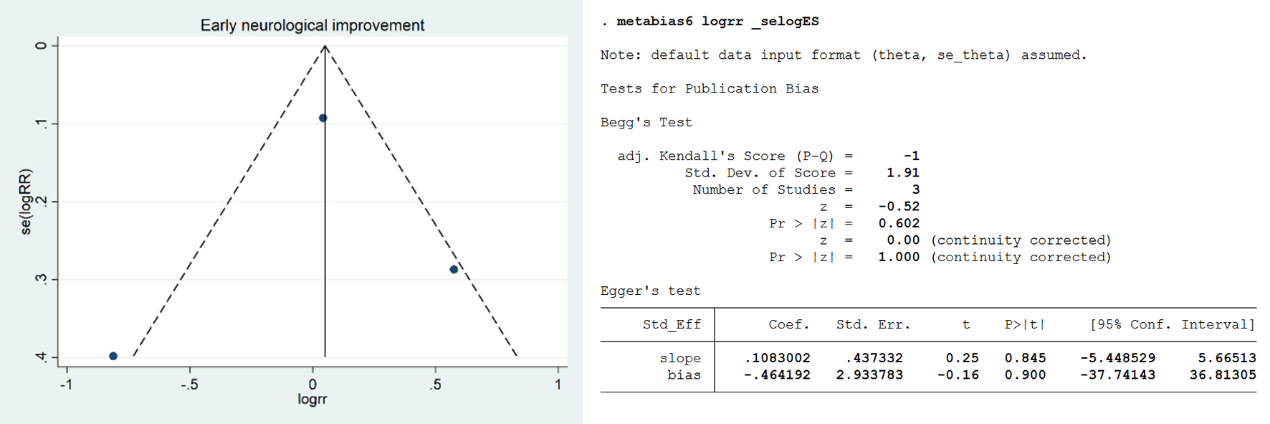
**

**FIGURE S11** Funnel plot and Egger’s and Begg’s tests for successful reperfusion

**
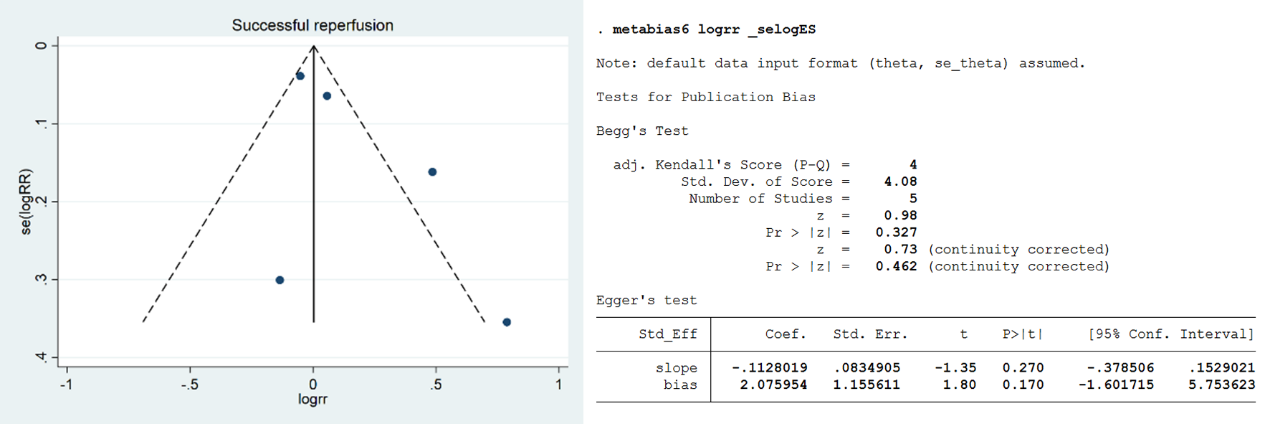
**

**FIGURE S12** Funnel plot and Egger’s and Begg’s tests for any parenchymal hematoma

**
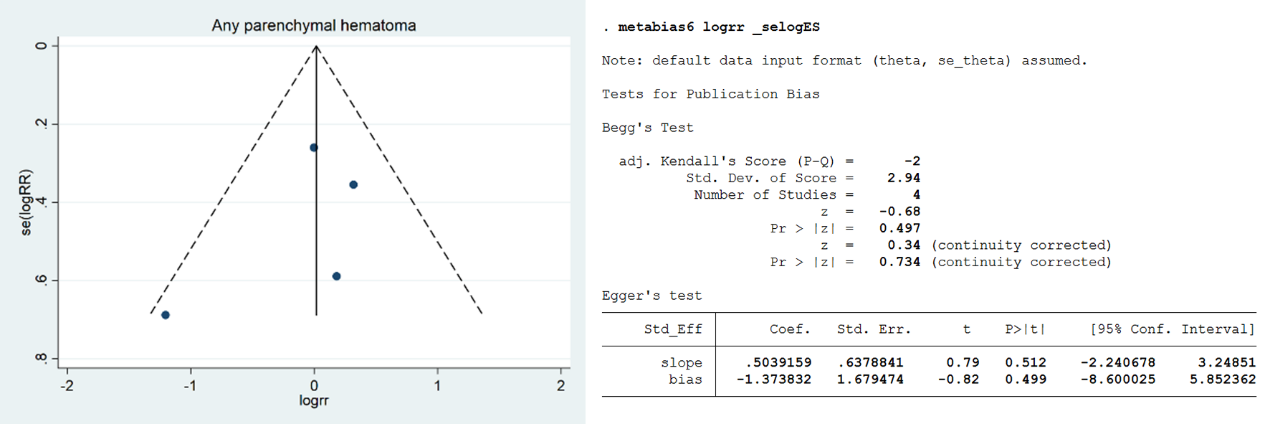
**

**FIGURE S13** Funnel plot and Egger’s and Begg’s tests for symptomatic intracranial hemorrhage

**
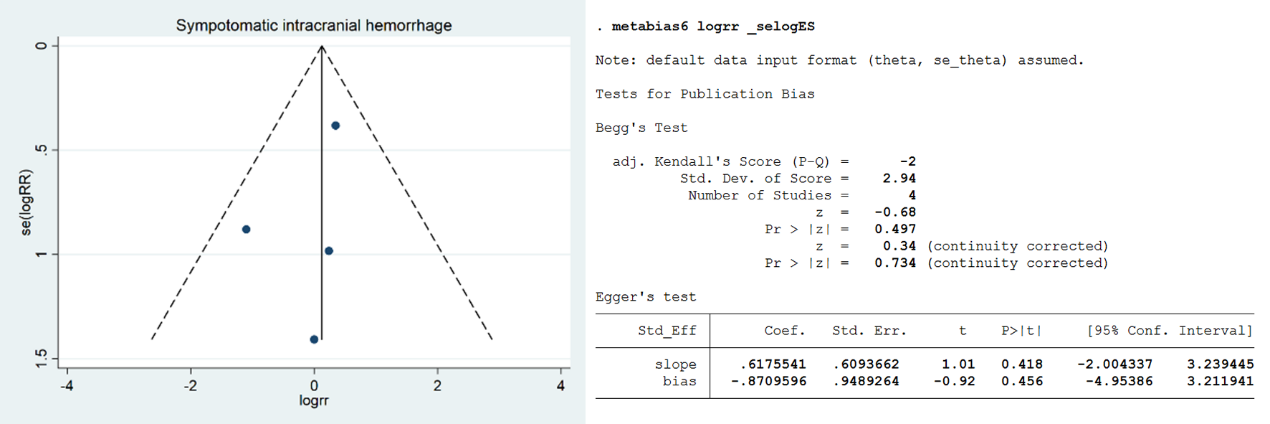
**

**FIGURE S14** Funnel plot and Egger’s and Begg’s tests for 3-month mortality


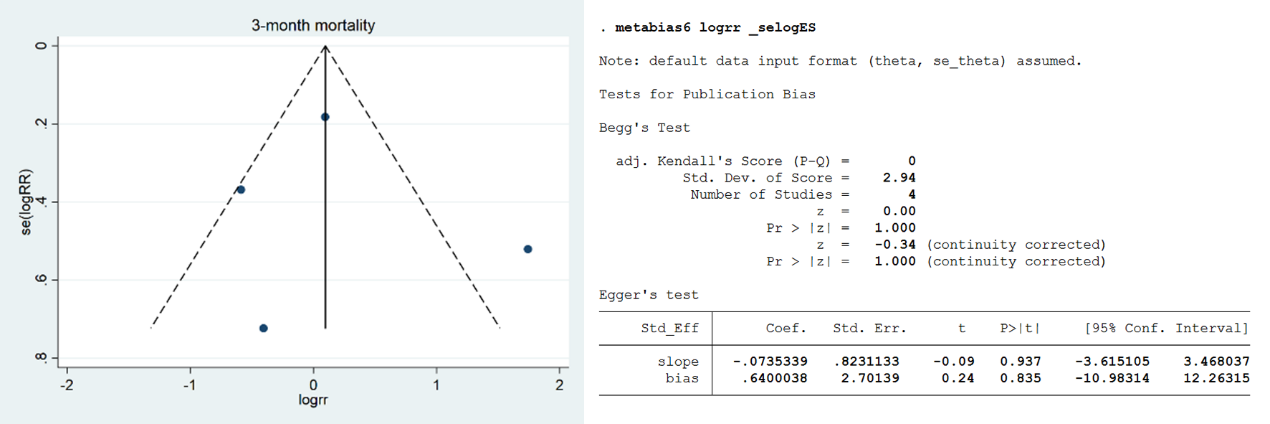

Supplement: Supplementary file 2 [file Table_2.DOCX]
