# Supplementary material for: Tenecteplase versus alteplase for intravenous thrombolysis of acute ischemic stroke patients with large-vessel occlusion: a systematic review and meta-analysis
Source: Front Neurol. 2025 Mar 19;16:1487711. doi: 10.3389/fneur.2025.1487711 (PMC11963696; doi:10.3389/fneur.2025.1487711)

**Supplemental figures to tenecteplase versus alteplase for intravenous thrombolysis of acute ischemic stroke patients with large vessel occlusion: a systematic review and meta-analysis**

**Forset plots without Australian TNK and ATTEST+Australian TNK trial**

**FIGURE S15** Forest plot for excellent neurological recovery


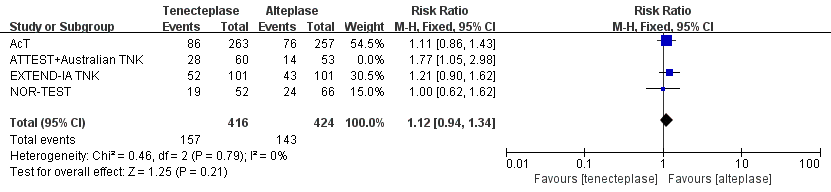


**FIGURE S16** Forest plot of good neurological recovery

*
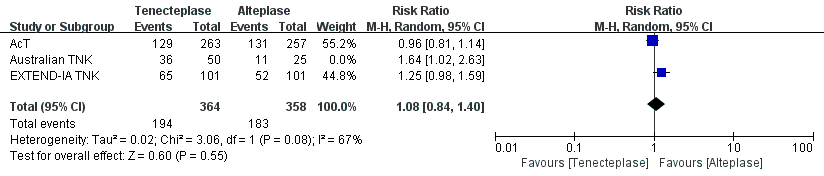
*

**FIGURE S17** Forest plot of early neurological improvement


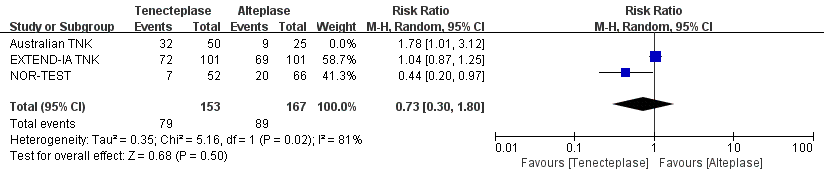


**FIGURE S18** Forest plot of successful reperfusion


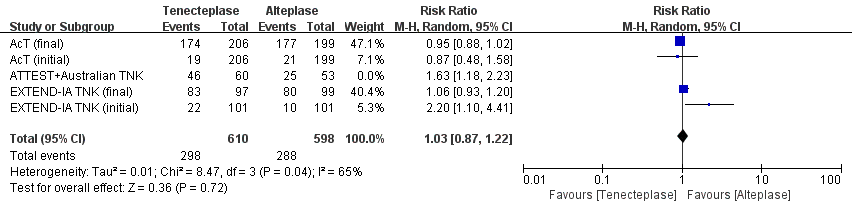


**FIGURE S19** Forest plot of any parenchymal hemotoma


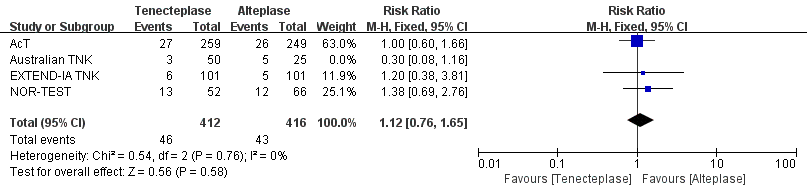


**FIGURE S20** Forest plot of symptomatic intracranial hemorrhage


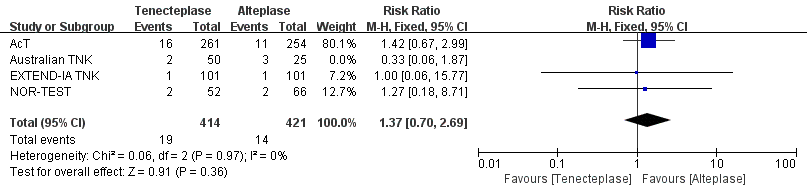


**FIGURE S21** Forest plot of 3-month mortality


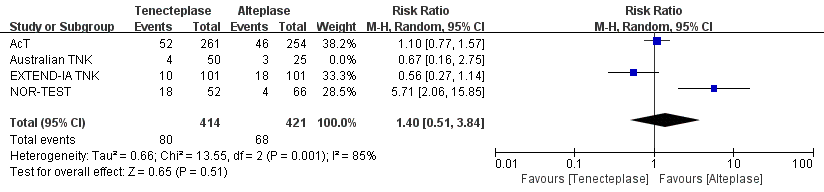

Supplement: Supplementary file 3 [file Table_3.DOCX]
