# Supplementary material for: Tenecteplase versus alteplase for intravenous thrombolysis of acute ischemic stroke patients with large-vessel occlusion: a systematic review and meta-analysis
Source: Front Neurol. 2025 Mar 19;16:1487711. doi: 10.3389/fneur.2025.1487711 (PMC11963696; doi:10.3389/fneur.2025.1487711)
Supplement: Supplementary file 4 [file Data_Sheet_1.docx]

Appendix S1: Search Strategy

Pubmed

**(((("Tenecteplase"[Mesh]) OR ((Metalyse[Title/Abstract]) OR (TNKase[Title/Abstract]))) AND (("Tissue Plasminogen Activator"[Mesh]) OR ((Plasminogen Activator, Tissue[Title/Abstract]) OR (Tissue Activator D-44[Title/Abstract]) OR (Tissue Activator D 44[Title/Abstract]) OR (Tisokinase[Title/Abstract]) OR (Tissue-Type Plasminogen Activator[Title/Abstract]) OR (Tissue Type Plasminogen Activator[Title/Abstract]) OR (TTPA[Title/Abstract]) OR (T-Plasminogen Activator[Title/Abstract]) OR (T Plasminogen Activator[Title/Abstract]) OR (Alteplase[Title/Abstract]) OR (Plasminogen Activator, Tissue-Type[Title/Abstract]) OR (Plasminogen Activator, Tissue Type[Title/Abstract]) OR (Activase[Title/Abstract]) OR (Actilyse[Title/Abstract]) OR (rt-PA[Title/Abstract]) OR (rt PA[Title/Abstract]) OR (rtPA[Title/Abstract])))) AND (((((("Stroke"[Mesh]) OR ((((((((((((((((((((((((((((Cerebral Strokes[Title/Abstract]) OR (Cerebral Stroke[Title/Abstract])) OR (Vascular Accident, Brain[Title/Abstract])) OR (Strokes[Title/Abstract])) OR (Apoplexy, Cerebrovascular[Title/Abstract])) OR (Apoplexy[Title/Abstract])) OR (Cerebrovascular Apoplexy[Title/Abstract])) OR (Cerebrovascular Stroke[Title/Abstract])) OR (Stroke, Cerebrovascular[Title/Abstract])) OR (Cerebrovascular Accident[Title/Abstract])) OR (Brain Vascular Accident[Title/Abstract])) OR (Cerebrovascular Accidents[Title/Abstract])) OR (Brain Vascular Accidents[Title/Abstract])) OR (Cerebrovascular Strokes[Title/Abstract])) OR (Stroke, Cerebral[Title/Abstract])) OR (CVAs (Cerebrovascular Accident[Title/Abstract]))) OR (Strokes, Cerebrovascular[Title/Abstract])) OR (CVA (Cerebrovascular Accident[Title/Abstract]))) OR (Vascular Accidents, Brain[Title/Abstract])) OR (Strokes, Cerebral[Title/Abstract])) OR (Cerebrovascular Accidents, Acute[Title/Abstract])) OR (Cerebrovascular Accident, Acute[Title/Abstract])) OR (Acute Strokes[Title/Abstract])) OR (Strokes, Acute[Title/Abstract])) OR (Acute Stroke[Title/Abstract])) OR (Acute Cerebrovascular Accident[Title/Abstract])) OR (Acute Cerebrovascular Accidents[Title/Abstract])) OR (Stroke, Acute[Title/Abstract]))) OR ("Cerebral Infarction"[Mesh])) OR (((((((((((((((((((((((((Posterior Choroidal Artery Infarction[Title/Abstract]) OR (Infarctions, Cerebral[Title/Abstract])) OR (Cerebral Infarctions[Title/Abstract])) OR (Cerebral Infarct[Title/Abstract])) OR (Infarction, Cerebral[Title/Abstract])) OR (Cerebral Infarcts[Title/Abstract])) OR (Infarct, Cerebral[Title/Abstract])) OR (Infarcts, Cerebral[Title/Abstract])) OR (Subcortical Infarction[Title/Abstract])) OR (Subcortical Infarctions[Title/Abstract])) OR (Infarctions, Subcortical[Title/Abstract])) OR (Infarction, Subcortical[Title/Abstract])) OR (Anterior Choroidal Artery Infarction[Title/Abstract])) OR (Cerebral Infarction, Right Hemisphere[Title/Abstract])) OR (Cerebral, Right Hemisphere, Infarction[Title/Abstract])) OR (Infarction, Right Hemisphere, Cerebral[Title/Abstract])) OR (Right Hemisphere, Infarction, Cerebral[Title/Abstract])) OR (Right Hemisphere, Cerebral Infarction[Title/Abstract])) OR (Infarction, Cerebral, Right Hemisphere[Title/Abstract])) OR (Cerebral Infarction, Left Hemisphere[Title/Abstract])) OR (Left Hemisphere, Infarction, Cerebral[Title/Abstract])) OR (Left Hemisphere, Cerebral Infarction[Title/Abstract])) OR (Infarction, Cerebral, Left Hemisphere[Title/Abstract])) OR (Infarction, Left Hemisphere, Cerebral[Title/Abstract])) OR (Cerebral, Left Hemisphere, Infarction[Title/Abstract]))) OR ("Brain Ischemia"[Mesh])) OR (((((((((Ischemias, Cerebral[Title/Abstract]) OR (Cerebral Ischemia[Title/Abstract])) OR (Ischemia, Cerebral[Title/Abstract])) OR (Cerebral Ischemias[Title/Abstract])) OR (Ischemic Encephalopathies[Title/Abstract])) OR (Ischemia, Brain[Title/Abstract])) OR (Brain Ischemias[Title/Abstract])) OR (Encephalopathy, Ischemic[Title/Abstract])) OR (Ischemic Encephalopathy[Title/Abstract])))) AND ("Randomized Controlled Trial" [Publication Type])**

EMBASE

**('cerebrovascular accident'/exp OR 'cerebrovascular accident' OR 'accident, cerebrovascular':ab,ti,kw OR 'acute cerebrovascular lesion':ab,ti,kw OR 'acute focal cerebral vasculopathy':ab,ti,kw OR 'acute stroke':ab,ti,kw OR 'apoplectic stroke':ab,ti,kw OR 'apoplexia':ab,ti,kw OR 'apoplexy':ab,ti,kw OR 'blood flow disturbance, brain':ab,ti,kw OR 'brain accident':ab,ti,kw OR 'brain attack':ab,ti,kw OR 'brain blood flow disturbance':ab,ti,kw OR 'brain insult':ab,ti,kw OR 'brain insultus':ab,ti,kw OR 'brain vascular accident':ab,ti,kw OR 'cerebral apoplexia':ab,ti,kw OR 'cerebral insult':ab,ti,kw OR 'cerebral stroke':ab,ti,kw OR 'cerebral vascular accident':ab,ti,kw OR 'cerebral vascular insufficiency':ab,ti,kw OR 'cerebro vascular accident':ab,ti,kw OR 'cerebrovascular arrest':ab,ti,kw OR 'cerebrovascular failure':ab,ti,kw OR 'cerebrovascular injury':ab,ti,kw OR 'cerebrovascular insufficiency':ab,ti,kw OR 'cerebrovascular insult':ab,ti,kw OR 'cerebrum vascular accident':ab,ti,kw OR 'cryptogenic stroke':ab,ti,kw OR 'cva':ab,ti,kw OR 'insultus cerebralis':ab,ti,kw OR 'ischaemic seizure':ab,ti,kw OR 'ischemic seizure':ab,ti,kw OR 'stroke':ab,ti,kw OR 'thrombotic stroke':ab,ti,kw OR 'cerebrovascular accident':ab,ti,kw OR 'brain infarction'/exp OR 'brain infarction' OR 'brain cortex infarct':ab,ti,kw OR 'brain cortex infarction':ab,ti,kw OR 'brain infarct':ab,ti,kw OR 'cerebral cortex infarct':ab,ti,kw OR 'cerebral cortex infarction':ab,ti,kw OR 'cerebral infarct':ab,ti,kw OR 'cerebral infarction':ab,ti,kw OR 'cerebrovascular infarct':ab,ti,kw OR 'cortical infarct':ab,ti,kw OR 'cerebrovascular infarction':ab,ti,kw OR 'cortical infarction':ab,ti,kw OR 'hemisphere infarct':ab,ti,kw OR 'hemisphere infarction':ab,ti,kw OR 'hemispheric infarct':ab,ti,kw OR 'hemispheric infarction':ab,ti,kw OR 'infarction, brain':ab,ti,kw OR 'silent brain infarction':ab,ti,kw OR 'brain infarction':ab,ti,kw OR 'brain ischemia'/exp OR 'brain ischemia' OR 'acute brain ischaemia':ab,ti,kw OR 'acute brain ischemia':ab,ti,kw OR 'brain arterial insufficiency':ab,ti,kw OR 'brain circulation disorder':ab,ti,kw OR 'brain ischaemia':ab,ti,kw OR 'cerebral blood circulation disorder':ab,ti,kw OR 'cerebral blood flow disorder':ab,ti,kw OR 'cerebral circulation disorder':ab,ti,kw OR 'cerebral circulatory disorder':ab,ti,kw OR 'cerebral ischaemia':ab,ti,kw OR 'cerebral ischemia':ab,ti,kw OR 'cerebrovascular circulation disorder':ab,ti,kw OR 'cerebrovascular ischaemia':ab,ti,kw OR 'cerebrovascular ischemia':ab,ti,kw OR 'ischaemia cerebri':ab,ti,kw OR 'ischaemic brain disease':ab,ti,kw OR 'ischaemic encephalopathy':ab,ti,kw OR 'ischemia cerebri':ab,ti,kw OR 'ischemic brain disease':ab,ti,kw OR 'ischemic encephalopathy':ab,ti,kw OR 'neural ischaemia':ab,ti,kw OR 'neural ischemia':ab,ti,kw OR 'brain ischemia':ab,ti,kw) AND ('tenecteplase'/exp OR 'tenecteplase' OR 'metalyse':ab,ti,kw OR 'r tpr 012':ab,ti,kw OR 'rg 3625':ab,ti,kw OR 'rg3625':ab,ti,kw OR 'rtpr012':ab,ti,kw OR 'tenecterel':ab,ti,kw OR 'tnk tpa':ab,ti,kw OR 'tenecteplase':ab,ti,kw OR 'tnkase':ab,ti,kw) AND ('alteplase'/exp OR 'alteplase' OR 'actilyse':ab,ti,kw OR 'activacin':ab,ti,kw OR 'activase':ab,ti,kw OR 'altelyse':ab,ti,kw OR 'atlepase':ab,ti,kw OR 'cathflo activase':ab,ti,kw OR 'g 11021':ab,ti,kw OR 'g 11044':ab,ti,kw OR 'g 11035':ab,ti,kw OR 'g11021':ab,ti,kw OR 'g11035':ab,ti,kw OR 'g11044':ab,ti,kw OR 'gmk 527':ab,ti,kw OR 'gmk527':ab,ti,kw OR 'grtpa':ab,ti,kw OR 'ly 210825':ab,ti,kw OR 'ly210825':ab,ti,kw OR 'mmr 701':ab,ti,kw OR 'mmr701':ab,ti,kw OR 'recombinant tissue plasminogen activator':ab,ti,kw OR 'recombinant tissue type plasminogen activator':ab,ti,kw OR 'revelise':ab,ti,kw OR 'sk and f 103796 i':ab,ti,kw OR 'td 2061':ab,ti,kw OR 'td2061':ab,ti,kw OR 'tissue plasminogen activator, recombinant':ab,ti,kw OR 'alteplase':ab,ti,kw) AND ('randomized controlled trial'/exp OR 'randomized controlled trial' OR 'controlled trial, randomized':ab,ti,kw OR 'randomised controlled study':ab,ti,kw OR 'randomised controlled trial':ab,ti,kw OR 'randomized controlled study':ab,ti,kw OR 'trial, randomized controlled':ab,ti,kw OR 'randomized controlled trial':ab,ti,kw)**

Cochrane Library

#1 MeSH descriptor: [Tenecteplase] explode all trees 201

#2 (Metalyse):ti,ab,kw OR (TNKase):ti,ab,kw (Word variations have been searched) 28

#3 #1 or #2 223

#4 MeSH descriptor: [Tissue Plasminogen Activator] explode all trees 2180

#5 (Actilyse):ti,ab,kw OR (Lysatec rtPA):ti,ab,kw OR (Lysatec rt PA):ti,ab,kw OR (Lysatec rt-PA):ti,ab,kw OR (Activase):ti,ab,kw OR (T-Plasminogen Activator):ti,ab,kw OR (Alteplase):ti,ab,kw OR (Tisokinase):ti,ab,kw OR (Plasminogen Activator, Tissue):ti,ab,kw OR (TTPA):ti,ab,kw OR (Tissue-Type Plasminogen Activator):ti,ab,kw OR (Plasminogen Activator, Tissue-Type):ti,ab,kw OR (Tissue Activator D-44):ti,ab,kw OR (T Plasminogen Activator):ti,ab,kw OR (Tissue Activator D 44):ti,ab,kw OR (Plasminogen Activator, Tissue Type):ti,ab,kw OR (Tissue Type Plasminogen Activator):ti,ab,kw 4946

#6 #4 or #5 4974

#7 MeSH descriptor: [Stroke] explode all trees 15355

#8 (Cerebral Strokes):ti,ab,kw OR (Cerebral Stroke):ti,ab,kw OR (Vascular Accident, Brain):ti,ab,kw OR (Strokes):ti,ab,kw OR (Apoplexy):ti,ab,kw OR (Apoplexy, Cerebrovascular):ti,ab,kw OR (Cerebrovascular Apoplexy):ti,ab,kw OR (Cerebrovascular Stroke):ti,ab,kw OR (Stroke, Cerebrovascular):ti,ab,kw OR (Cerebrovascular Accident):ti,ab,kw OR (Brain Vascular Accident):ti,ab,kw OR (Cerebrovascular Accidents):ti,ab,kw OR (Brain Vascular Accidents):ti,ab,kw OR (Cerebrovascular Strokes):ti,ab,kw OR (Stroke, Cerebral):ti,ab,kw OR (CVAs (Cerebrovascular Accident)):ti,ab,kw OR (Strokes, Cerebrovascular ):ti,ab,kw OR (Vascular Accidents, Brain):ti,ab,kw OR (CVA (Cerebrovascular Accident)):ti,ab,kw OR (Strokes, Cerebral):ti,ab,kw OR (Cerebrovascular Accidents, Acute):ti,ab,kw OR (Cerebrovascular Accident, Acute):ti,ab,kw OR (Strokes, Acute):ti,ab,kw OR (Acute Strokes):ti,ab,kw OR (Acute Stroke):ti,ab,kw OR (Acute Cerebrovascular Accident):ti,ab,kw OR (Acute Cerebrovascular Accidents):ti,ab,kw OR (Stroke, Acute ):ti,ab,kw 39792

#9 MeSH descriptor: [Cerebral Infarction] explode all trees 1522

#10 (Posterior Choroidal Artery Infarction ):ti,ab,kw OR (Infarctions, Cerebral ):ti,ab,kw OR (Cerebral Infarctions ):ti,ab,kw OR (Cerebral Infarct ):ti,ab,kw OR (Infarction, Cerebral ):ti,ab,kw OR (Cerebral Infarcts ):ti,ab,kw OR (Infarct, Cerebral ):ti,ab,kw OR (Infarcts, Cerebral):ti,ab,kw OR (Subcortical Infarction):ti,ab,kw OR (Subcortical Infarctions ):ti,ab,kw OR (Infarctions, Subcortical ):ti,ab,kw OR (Infarction, Subcortical ):ti,ab,kw OR (Anterior Choroidal Artery Infarction ):ti,ab,kw OR (Cerebral Infarction, Right Hemisphere ):ti,ab,kw OR (Cerebral, Right Hemisphere, Infarction ):ti,ab,kw OR (Infarction, Right Hemisphere, Cerebral ):ti,ab,kw OR (Right Hemisphere, Infarction, Cerebral ):ti,ab,kw OR (Right Hemisphere, Cerebral Infarction ):ti,ab,kw OR (Infarction, Cerebral, Right Hemisphere):ti,ab,kw OR (Cerebral Infarction, Left Hemisphere):ti,ab,kw OR (Left Hemisphere, Infarction, Cerebral):ti,ab,kw OR ( Left Hemisphere, Cerebral Infarction):ti,ab,kw OR (Infarction, Cerebral, Left Hemisphere):ti,ab,kw OR (Infarction, Left Hemisphere, Cerebral):ti,ab,kw OR (Cerebral, Left Hemisphere, Infarction):ti,ab,kw 6759

#11 MeSH descriptor: [Brain Ischemia] explode all trees 6043

#12 (Ischemias, Cerebral ):ti,ab,kw OR (Cerebral Ischemia):ti,ab,kw OR (Ischemia, Cerebral):ti,ab,kw OR (Cerebral Ischemias):ti,ab,kw OR (Ischemic Encephalopathies):ti,ab,kw OR (Brain Ischemias):ti,ab,kw OR (Ischemia, Brain):ti,ab,kw OR (Encephalopathy, Ischemic):ti,ab,kw OR (Ischemic Encephalopathy):ti,ab,kw 9711

#13 #7 or #8 or #9 or #10 or #11 or #12 52235

#14 #3 and #6 and #13 109

Web of Science

#1 ((TS=(tenecteplase)) OR TS=(Metalyse)) OR TS=(TNKase) and Preprint Citation Index (Exclude – Database)

#2 TS=(stroke) OR TS=(Cerebral Strokes) OR TS=(Cerebral Stroke) OR TS=(Vascular Accident, Brain) OR TS=(Strokes) OR TS=(Apoplexy, Cerebrovascular) OR TS=(Apoplexy) OR TS=(Cerebrovascular Apoplexy) OR TS=(Cerebrovascular Stroke) OR TS=(Stroke, Cerebrovascular) OR TS=(Cerebrovascular Accident) OR TS=(Brain Vascular Accident) OR TS=(Cerebrovascular Accidents) OR TS=(Brain Vascular Accidents) OR TS=(Cerebrovascular Strokes) OR TS=(Stroke, Cerebral) OR TS=(CVAs (Cerebrovascular Accident)) OR TS=(Strokes, Cerebrovascular ) OR TS=(CVA (Cerebrovascular Accident)) OR TS=(Vascular Accidents, Brain) OR TS=(Strokes, Cerebral) OR TS=(Cerebrovascular Accidents, Acute) OR TS=(Cerebrovascular Accident, Acute) OR TS=(Acute Strokes) OR TS=(Strokes, Acute) OR TS=(Acute Stroke) OR TS=(Acute Cerebrovascular Accident) OR TS=(Acute Cerebrovascular Accidents) OR TS=(Stroke, Acute ) and Preprint Citation Index (Exclude – Database)

#3 TS=(cerebral infarction) OR TS=(Posterior Choroidal Artery Infarction ) OR TS=(Infarctions, Cerebral ) OR TS=(Cerebral Infarctions ) OR TS=(Cerebral Infarct ) OR TS=(Infarction, Cerebral ) OR TS=(Cerebral Infarcts ) OR TS=(Infarct, Cerebral ) OR TS=(Infarcts, Cerebral) OR TS=(Subcortical Infarctions ) OR TS=(Subcortical Infarction) OR TS=(Infarctions, Subcortical ) OR TS=(Infarction, Subcortical ) OR TS=(Anterior Choroidal Artery Infarction ) OR TS=(Cerebral Infarction, Right Hemisphere ) OR TS=(Cerebral, Right Hemisphere, Infarction ) OR TS=(Infarction, Right Hemisphere, Cerebral ) OR TS=(Right Hemisphere, Infarction, Cerebral ) OR TS=(Right Hemisphere, Cerebral Infarction ) OR TS=(Infarction, Cerebral, Right Hemisphere) OR TS=(Cerebral Infarction, Left Hemisphere) OR TS=(Left Hemisphere, Infarction, Cerebral) OR TS=( Left Hemisphere, Cerebral Infarction) OR TS=(Infarction, Cerebral, Left Hemisphere) OR TS=(Infarction, Left Hemisphere, Cerebral) OR TS=(Cerebral, Left Hemisphere, Infarction) and Preprint Citation Index (Exclude – Database)

#4 TS=(brain ischemia) OR TS=(Cerebral Ischemia) OR TS=( Ischemias, Cerebral ) OR TS=(Ischemia, Cerebral) OR TS=(Cerebral Ischemias) OR TS=(Ischemic Encephalopathies) OR TS=(Ischemia, Brain) OR TS=(Brain Ischemias) OR TS=(Ischemic Encephalopathy) OR TS=(Encephalopathy, Ischemic) and Preprint Citation Index (Exclude – Database)

#5 TS=(Tissue Plasminogen Activator) OR TS=( Actilyse) OR TS=(Lysatec rtPA) OR TS=(Lysatec rt-PA) OR TS=(Lysatec rt PA) OR TS=(Activase) OR TS=(T-Plasminogen Activator) OR TS=(Tisokinase) OR TS=(Plasminogen Activator, Tissue) OR TS=(TTPA) OR TS=(Tissue-Type Plasminogen Activator) OR TS=(Plasminogen Activator, Tissue-Type) OR TS=(Tissue Activator D-44) OR TS=(Tissue Activator D 44) OR TS=(T Plasminogen Activator) OR TS=(Plasminogen Activator, Tissue Type) OR TS=(Tissue Type Plasminogen Activator) OR TS=(alteplase) and Preprint Citation Index (Exclude – Database)

#6 TS=(Randomized Controlled Trial) OR TS=(controlled trial, randomized) OR TS=(randomised controlled study) OR TS=(randomised controlled trial) OR TS=(randomized controlled study) OR TS=(trial, randomized controlled) OR TS=(randomized controlled trial) and Preprint Citation Index (Exclude – Database)

#7 #2 OR #3 OR #4 and Preprint Citation Index (Exclude – Database)

#8 #1 AND #5 AND #6 AND #7 and Preprint Citation Index (Exclude – Database)

ClinicalTrials.gov

**(((("Tenecteplase"[Mesh]) OR ((Metalyse[Title/Abstract]) OR (TNKase[Title/Abstract]))) AND (("Tissue Plasminogen Activator"[Mesh]) OR ((Plasminogen Activator, Tissue[Title/Abstract]) OR (Tissue Activator D-44[Title/Abstract]) OR (Tissue Activator D 44[Title/Abstract]) OR (Tisokinase[Title/Abstract]) OR (Tissue-Type Plasminogen Activator[Title/Abstract]) OR (Tissue Type Plasminogen Activator[Title/Abstract]) OR (TTPA[Title/Abstract]) OR (T-Plasminogen Activator[Title/Abstract]) OR (T Plasminogen Activator[Title/Abstract]) OR (Alteplase[Title/Abstract]) OR (Plasminogen Activator, Tissue-Type[Title/Abstract]) OR (Plasminogen Activator, Tissue Type[Title/Abstract]) OR (Activase[Title/Abstract]) OR (Actilyse[Title/Abstract]) OR (rt-PA[Title/Abstract]) OR (rt PA[Title/Abstract]) OR (rtPA[Title/Abstract])))) AND (((((("Stroke"[Mesh]) OR ((((((((((((((((((((((((((((Cerebral Strokes[Title/Abstract]) OR (Cerebral Stroke[Title/Abstract])) OR (Vascular Accident, Brain[Title/Abstract])) OR (Strokes[Title/Abstract])) OR (Apoplexy, Cerebrovascular[Title/Abstract])) OR (Apoplexy[Title/Abstract])) OR (Cerebrovascular Apoplexy[Title/Abstract])) OR (Cerebrovascular Stroke[Title/Abstract])) OR (Stroke, Cerebrovascular[Title/Abstract])) OR (Cerebrovascular Accident[Title/Abstract])) OR (Brain Vascular Accident[Title/Abstract])) OR (Cerebrovascular Accidents[Title/Abstract])) OR (Brain Vascular Accidents[Title/Abstract])) OR (Cerebrovascular Strokes[Title/Abstract])) OR (Stroke, Cerebral[Title/Abstract])) OR (CVAs (Cerebrovascular Accident[Title/Abstract]))) OR (Strokes, Cerebrovascular[Title/Abstract])) OR (CVA (Cerebrovascular Accident[Title/Abstract]))) OR (Vascular Accidents, Brain[Title/Abstract])) OR (Strokes, Cerebral[Title/Abstract])) OR (Cerebrovascular Accidents, Acute[Title/Abstract])) OR (Cerebrovascular Accident, Acute[Title/Abstract])) OR (Acute Strokes[Title/Abstract])) OR (Strokes, Acute[Title/Abstract])) OR (Acute Stroke[Title/Abstract])) OR (Acute Cerebrovascular Accident[Title/Abstract])) OR (Acute Cerebrovascular Accidents[Title/Abstract])) OR (Stroke, Acute[Title/Abstract]))) OR ("Cerebral Infarction"[Mesh])) OR (((((((((((((((((((((((((Posterior Choroidal Artery Infarction[Title/Abstract]) OR (Infarctions, Cerebral[Title/Abstract])) OR (Cerebral Infarctions[Title/Abstract])) OR (Cerebral Infarct[Title/Abstract])) OR (Infarction, Cerebral[Title/Abstract])) OR (Cerebral Infarcts[Title/Abstract])) OR (Infarct, Cerebral[Title/Abstract])) OR (Infarcts, Cerebral[Title/Abstract])) OR (Subcortical Infarction[Title/Abstract])) OR (Subcortical Infarctions[Title/Abstract])) OR (Infarctions, Subcortical[Title/Abstract])) OR (Infarction, Subcortical[Title/Abstract])) OR (Anterior Choroidal Artery Infarction[Title/Abstract])) OR (Cerebral Infarction, Right Hemisphere[Title/Abstract])) OR (Cerebral, Right Hemisphere, Infarction[Title/Abstract])) OR (Infarction, Right Hemisphere, Cerebral[Title/Abstract])) OR (Right Hemisphere, Infarction, Cerebral[Title/Abstract])) OR (Right Hemisphere, Cerebral Infarction[Title/Abstract])) OR (Infarction, Cerebral, Right Hemisphere[Title/Abstract])) OR (Cerebral Infarction, Left Hemisphere[Title/Abstract])) OR (Left Hemisphere, Infarction, Cerebral[Title/Abstract])) OR (Left Hemisphere, Cerebral Infarction[Title/Abstract])) OR (Infarction, Cerebral, Left Hemisphere[Title/Abstract])) OR (Infarction, Left Hemisphere, Cerebral[Title/Abstract])) OR (Cerebral, Left Hemisphere, Infarction[Title/Abstract]))) OR ("Brain Ischemia"[Mesh])) OR (((((((((Ischemias, Cerebral[Title/Abstract]) OR (Cerebral Ischemia[Title/Abstract])) OR (Ischemia, Cerebral[Title/Abstract])) OR (Cerebral Ischemias[Title/Abstract])) OR (Ischemic Encephalopathies[Title/Abstract])) OR (Ischemia, Brain[Title/Abstract])) OR (Brain Ischemias[Title/Abstract])) OR (Encephalopathy, Ischemic[Title/Abstract])) OR (Ischemic Encephalopathy[Title/Abstract])))) AND ("Randomized Controlled Trial" [Publication Type])**
